# Molecular Simulation of the Adsorption Separation of Acidic Natural Gas Contaminants with Zeolites

**DOI:** 10.3390/nano16020131

**Published:** 2026-01-19

**Authors:** Tamás Kristóf, Levente Fodor

**Affiliations:** Center for Natural Sciences, University of Pannonia, Egyetem str. 10, H-8200 Veszprém, Hungary

**Keywords:** zeolites, carbon dioxide separation, molecular simulation

## Abstract

From an energetic, economic and environmental perspective, the selective removal of carbon dioxide and hydrogen sulfide from industrial natural gas streams is crucial. For this purpose, adsorption separation using nanoporous zeolites composed solely of silicon and oxygen atoms is a promising and environmentally friendly alternative to conventional adsorption and absorption processes. In this study, the adsorption of binary and ternary gas mixtures containing carbon dioxide, methane and/or hydrogen sulfide was examined with more than 100 different pure silica zeolites using atomic-resolution grand canonical Monte Carlo simulations. The IZA database was searched primarily for zeolites that could potentially be used to separate carbon dioxide from methane. However, many of the frameworks found were also suitable for the selective separation of hydrogen sulfide. The dependence of the calculated selectivities on pressure, temperature and gas composition was investigated, and a multi-step adsorption test was also performed with the zeolites showing the best performance. An empirical relationship was observed between certain structural parameters and the preference for binding carbon dioxide. This equation was then used to systematically screen a large database of theoretical zeolites. As a result, not only some IZA zeolites but also several theoretical zeolite structures were identified that strongly favor the adsorption of carbon dioxide over methane.

## 1. Introduction

Natural gas is a mixture of gases whose composition varies significantly depending on its place of origin. It typically contains over 95 mol% methane, smaller quantities of other hydrocarbons, and sometimes relatively high concentrations of carbon dioxide and hydrogen sulfide. These acidic gases reduce the quality and caloric value of natural gas, contribute to the degradation of various types of equipment. When released into the environment, carbon dioxide contributes to the global greenhouse effect. Strict limits are generally applied to processed gas products in industry; e.g., the hydrogen sulfide content must often be below 4 ppm. For the separation of carbon dioxide and hydrogen sulfide from natural gas, adsorption-based processes offer, due to their favorable selectivity and better energy efficiency, a promising alternative to traditional absorption-based purification techniques using a liquid solvent that selectively dissolves carbon dioxide and/or hydrogen sulfide. However, such adsorption separation is challenging, as both molecules to be separated are as small as methane molecules, and their acidic nature and good solubility in water can hinder their selective removal. Commonly used adsorbents include zeolites [1,2,3], metal–organic frameworks [4,5], activated carbon [6] and metal oxide materials [7].

Zeolites are currently receiving increasing attention in the field of industrial gas separation. These naturally occurring aluminosilicates are characterized by a well-defined pore structure, a large specific surface area, structural stability and regenerability. The central element of their structure is the tetrahedrally coordinated silicon (Si) atom, which can sometimes be replaced by an aluminum (Al) atom. Zeolites are extremely diverse in structure; the connection possibilities of their basic SiO_2_ units allow for an almost infinite number of combinations in the geometry of their cages and the topology of their pore systems. Naturally occurring or synthesized zeolites are listed in the IZA-SC (Structure Commission of the International Zeolite Association) database [1]. According to the database, there are more than 260 distinct, crystalline zeolite structures, each identified by a three-letter code. It is also important to note that a huge number of theoretical zeolites have been generated by computers (see PCOD database [8,9,10]). When assessing their potential adsorption capabilities, the aim is to also estimate, based on various energetic and structural considerations, which structures have a real chance of being synthesized in laboratories [11,12].

The adsorption properties of zeolites depend on their crystal structure and pore geometry, as well as their ability to form strong electrostatic interactions with the adsorbates. In the case of Al substitution, the negative charges generated within the lattice are neutralized by charge-compensating cations, which can significantly affect the adsorption properties depending on their type, size and location [1]. A recent review of the potential use of natural zeolites for carbon dioxide capture can be found in [13]. As pure silica zeolite frameworks are electrically neutral, their adsorption properties are solely determined by pore geometry, channel topology and adsorbate molecular size. These zeolites are more hydrophobic and thermally stable, and their separation performance is less sensitive to the moisture content.

With advances in materials science, the identification of potentially good adsorbents is, in addition to experimental testing, increasingly relying on theoretical methods. Methods based on classical molecular simulations are becoming increasingly popular as they allow adsorbents to be screened and compared systematically and relatively quickly and cost-effectively, even under extreme conditions. Recent studies have addressed the applicability of zeolites containing only Si and O atoms for the removal of acidic gases. In a comprehensive study [14], e.g., 386 different pure silica zeolites were analyzed using Monte Carlo simulations to reveal the adsorption properties of H_2_S-CH_4_ and H_2_S-C_2_H_6_ binary gas mixtures. The 16 most promising structures were then evaluated by simulating a five-component gas mixture (CH_4_, C_2_H_6_, CO_2_, N_2_, H_2_S) under real industrial conditions. The results clearly showed that optimal separation of impurity components depends on the composition of the gas mixture and the geometry of the zeolite pores. Another simulation study [15] identified 16 promising IZA zeolites for removing acidic pollutants from natural gas, and industrial pressure swing adsorption–desorption cycles were modeled with 3 of them. This research represents an important step forward in the integration of molecular simulation results into process modeling systems. In our simulation studies [16,17], we conducted in-depth research into the potential structures of idealized/optimized pure silica zeolites for separating hydrogen sulfide from light alkanes. All these studies emphasize the importance of eight-membered entry windows in zeolitic pores, which have been shown to be a critical limiting factor in adsorption. Smaller entry window sizes prevent the gas molecules under discussion from entering the framework. In the case of gas mixtures containing ethane and propane molecules, the selectivity of hydrogen sulfide towards the alkanes can be explained by the size effect. However, this is not valid for gas mixtures containing methane. In electrically neutral, pure silica frameworks, it is only the polar nature of the Si-O bonds that gives hydrogen sulfide, which contains more polar S-H bonds, an advantage over methane adsorption. While it was observed that pore size distribution could predict separation performance in these gas mixtures to a certain extent, the selectivity trends could not be fully explained by this property alone [16].

The aim of this work is to identify additional pure silica zeolite structures that have a sufficiently high adsorption capacity to selectively bind carbon dioxide and hydrogen sulfide from methane-containing gas mixtures, while absorbing only a minimal amount of methane. Atomic resolution grand canonical Monte Carlo simulations were applied for the investigations, using interaction models that demonstrate a high level of agreement with the experimental data found in the literature. This study involved simulating the adsorption properties of over 100 zeolite structures, with a primary focus on carbon dioxide and a secondary focus on hydrogen sulfide. Additionally, a structural analysis was performed to pre-select zeolites for the atomistic simulations. First, we examined zeolite structures in the IZA database [1], including both natural and synthetic frameworks (note that some of the IZA zeolites studied in this work are not currently available in pure silica form; they can potentially be obtained through further synthesis or post-treatment procedures). After analyzing the results, we screened databases containing theoretical zeolites that are likely to be synthesized in the future. In this way, we were able to identify zeolites promising for industrial applications from the initial large set of frameworks. In addition to the above-mentioned works, several other simulation studies have addressed carbon dioxide adsorption on zeolites (see, for example, [18]), but such a comprehensive investigation, which would include both available and theoretical pure silica zeolites, has not yet been conducted.

## 2. Materials and Methods

The grand canonical (fixed *µVT*) Monte Carlo simulation method is ideal for determining the equilibrium conditions of gas adsorption on a fixed-volume (*V*) solid adsorbent. The thermodynamic parameters of the adsorption phase can be specified by the temperature (*T*) and the chemical potential (*µ*) of the gas component to be adsorbed. *µ* is the chemical potential that also characterizes the contacting gas phase at temperature *T* and pressure *p* in thermodynamic equilibrium (therefore, it is sufficient to implicitly consider the gas phase as a material reservoir characterized only by the specified *µ* and *T* in the grand canonical simulation). For these simulations, the RASPA 2.0 simulation software package was used [19,20]. This software allows the gas phase pressure (or partial pressure in mixtures) to be specified directly, and automatically calculates the required chemical potential based on the Peng-Robinson equation of state.

The predictive power of adsorption simulations depends on the interaction models chosen for the given system. For zeolites, the widely accepted TraPPE force field [21], which applies additive Lennard-Jones (12-6) and Coulomb interactions to atom pairs, was used. This Lennard-Jones plus Coulomb interaction scheme was also used to describe the atomic interactions of the adsorbing molecules. Intramolecular interactions were neglected in all cases by fixing the experimentally or theoretically determined positions of the atoms.

A relatively new potential model [22] was chosen for hydrogen sulfide containing five interaction centers that had been tested in our previous study [16]. A remarkable feature of the model is that the non-bonding electron pairs of the sulfur atom are also represented by massless, virtual interaction sites that do not have Lennard-Jones interactions and contribute only as point charges to the description of the charge distribution of the molecule. To describe methane, we also selected a potential model [23] that had been tested and slightly modified in our previous study [16]. To describe carbon dioxide, we started from the TraPPE model [24] containing three atomic interaction centers. In this work, we tested the latter model by simulating the adsorption isotherm on zeolite CHA at 298 K, and we were able to reproduce the available experimental data [25] with satisfactory accuracy by adjusting the model parameters. During the fitting procedure, it was found that only the energy parameter of the Lennard-Jones potential for the carbon atom needed to be modified. This change did not directly affect the molecule’s Coulomb energy and resulted in an increase of less than 10% in the total Lennard-Jones energy of the interacting molecule. The parameters of the potential models used in the simulations are listed in Table 1.

For the grand canonical Monte Carlo simulations, structural pre-screening of the pure silica zeolites of the IZA database [1] was carried out using the PoreBlazer v4.0 software [26]. The PoreBlazer software was developed to rapidly map the structural properties of solid porous materials. It can determine the internal surface area and volume, the pore size distribution, as well as the so-called pore limiting diameter (PLD) and largest cavity diameter (LCD) of the pore system. The PLD parameter indicates the maximum diameter of a rigid sphere that can pass through the lattice in at least one direction along a continuous path. The LCD parameter, on the other hand, indicates the maximum accessible pore diameter along this path.

The atomistic simulations were started with the recording of adsorption isotherms for the pure gas components (CO_2_, H_2_S and CH_4_). These were determined for all zeolites identified by the PoreBlazer software as potentially suitable for the separation task. The adsorption properties with two- and three-component gas mixtures were then calculated and analyzed. The simulations were performed at two temperatures (298 K, 323 K), several pressures (100 kPa, 1000 kPa, 5000 kPa, etc.), and with different compositions.

The Monte Carlo simulations consisted of 100,000 cycles in total. Of these, 20,000 cycles were used to establish equilibrium, and 80,000 cycles were used for equilibrium sampling (the number of molecular moves in one cycle corresponded to the number of fluid particles present in the framework, but at least 20 steps had to be reached). Periodic boundary conditions were applied in all directions, and the long-range Coulomb interactions were calculated using Ewald summation [27]. A cutoff length of 1.4 nm was specified for the direct calculation of interactions (applying standard long-range correction for the Lennard-Jones interactions [28]). The size of the simulation box was set by multiplying the unit cell length parameters of the zeolite lattice, ensuring it was at least 2.8 nm in all directions. The probabilities of the various attempted particle moves (insertion of one particle per component into the framework, removal of a particle from the framework, displacement of a particle within the framework) were set to be identical. The combination of the Lennard-Jones parameters for mixed atom-atom interactions was based on the Lorentz-Berthelot rule.

The most important properties for evaluating the equilibrium adsorption performance of zeolites are the adsorption amount (*q*), selectivity (*α*) and heat of adsorption (Δ*H*_ads_). Selectivity is calculated by comparing the ratio of the adsorption amounts from a given gas mixture with the ratio of the mole fractions *y* in the gas mixture:

*α* = (*q*_CO_2__/*q*_CH_4__)/(*y*_CO_2__/*y*_CH_4__). (1)

Adsorption heat is a measure of the energy change that occurs when one mole of gas molecules binds to the zeolite lattice. Its value indicates the strength of the interaction between the lattice and the adsorbed molecule. In the case of rigid models, it can be calculated using the following approximate formula [19]:

Δ*H*_ads_ = <*U*_a_> − *RT*, (2)

where *R* is the universal gas constant and <*U*_a_> is the average potential energy of the adsorbed molecules.

## 3. Results and Discussion

Candidates were first selected from the pure silica zeolite frameworks with idealized/optimized structures in the IZA database [1] based on preliminary screening using the PoreBlazer software. The selection was guided by considerations from previous works [14,15,16]; we searched for zeolites with structural characteristics similar to those of zeolites found to selectively adsorb carbon dioxide or hydrogen sulfide from methane-containing gas mixtures. Table 2 shows some of the characteristics of the 37 IZA zeolites that were chosen for further analysis. Based on preliminary knowledge, structures with PLD values between 0.3 and 0.4 nm and LCD values between 0.4 and 0.6 nm were primarily included. However, for verification purposes, a few zeolites with different PLD or LCD values were also examined. The group contains zeolites ACO, APC, APD, BIK, CAS and CHA for reference, the adsorption properties of which from binary gas mixtures of methane and hydrogen sulfide, or methane and carbon dioxide, had already been studied by us [16], or by others [15]. We intended to gradually narrow down the group of zeolites under study, carrying out grand canonical simulations under an increasing number of conditions. The primary simulation selection was based on the adsorption isotherms of the pure gas components and mixture adsorption tests involving binary gas mixtures of methane and carbon dioxide (see Table 2). Zeolite MVY, e.g., was filtered out in this way, even though it appears to have suitable low PLD and LCD parameters; unlike most of the other zeolites in Table 2, its carbon dioxide adsorption coverage remains below 0.2 mol/kg even at 1000 kPa, and the adsorption of the other two components is always negligible.

We also used the PoreBlazer program to verify that practically all the adsorption sites for the studied zeolites are accessible from the outer entry points of the pores. This means that, in grand canonical simulations (which involve randomly placing and removing molecules), adsorption is unlikely to occur at framework positions that are inaccessible from the outside (which is consistent with real experiments).

### 3.1. Pure Components’ Isotherms

**Figure 1:**
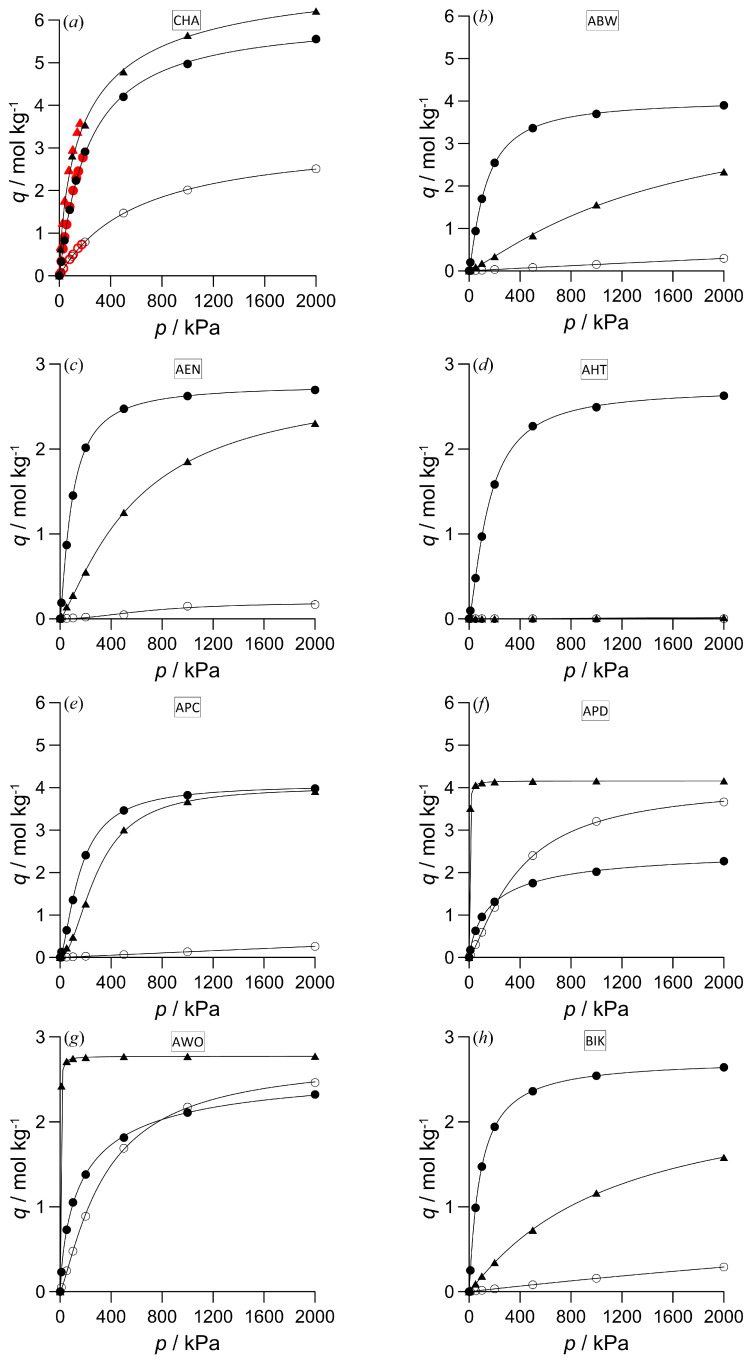

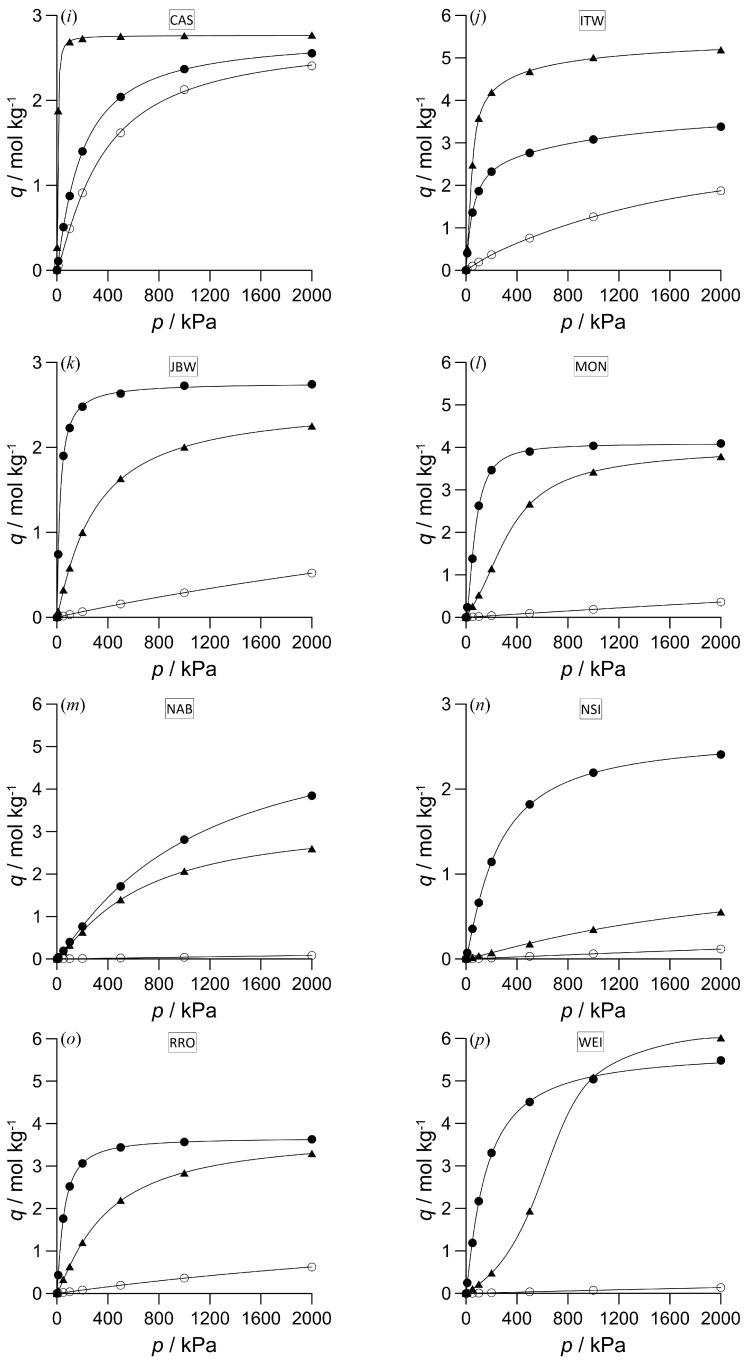
Equilibrium adsorption isotherms for pure carbon dioxide (●), hydrogen sulfide (▲) and methane (○) on the 16 selected IZA zeolites at 298 K. Symbols are the simulation results (statistical uncertainties do not exceed the symbol size) and lines are the fitted DSLF isotherm curves [29]. Experimental data on zeolite CHA [25] are indicated by red symbols.

Of the zeolites shown in Table 2, the pure components adsorption isotherms for 16 frameworks that showed the most favorable or interesting adsorption behavior are presented in Figure 1 (to facilitate comparison of the adsorption capacities of different zeolites, only two possible maximum values are set for the vertical axes). The equilibrium loading data points produced by the simulations could be accurately fitted using the Dual-Site Langmuir-Freundlich (DSLF) equilibrium adsorption model:

*q* = *q_sb_bp*^1/*nb*^/(1 + *bp*^1/*nb*^) + *q_sd_dp*^1/*nd*^/(1 + *dp*^1/*nd*^). (3)

In this six-parameter equation, *q_sb_* and *q_sd_* are the solid-phase saturation loadings, *b* and *d* are the affinity coefficients, *n_b_* and *n_d_* represent the ideal homogeneous surface deviations of the adsorbed component at adsorption sites of types 1 and 2, respectively, and *p* is the pressure [29]. Applying Equation (1) with pressure ratios instead of gas-phase mole fraction ratios to an isotherm data pair of two pure components often provides a good approximation of the selectivity that can be obtained from mixture adsorption simulations. However, significant deviations may occur at higher pressures due to competition between the molecules of the adsorbing gas components.

In the case of zeolite CHA (Figure 1a), the reliability of the potential models has been verified. This figure panel shows a high level of agreement between the simulations and the available experimental data [25]. The obtained adsorption isotherms can mostly be classified as IUPAC type I (Langmuir type), although adsorption on zeolites does not meet the conditions of an ideal Langmuir isotherm. Overall, the adsorption amounts of carbon dioxide and hydrogen sulfide are generally higher than those of methane, and the adsorption of the acidic substances alternates in dominance with these zeolites. Methane adsorption frequently follows a linear curve with a slight initial slope. This suggests that the interactions between the methane molecule being adsorbed and the framework are weaker, and that the degree of methane adsorption may increase further outside the range studied. Some isotherms for hydrogen sulfide reach saturation capacity very quickly (e.g., zeolites ACO, ATV, AWO, and CAS); a notable exception is the isotherm with zeolite WEI, where the curve is convex in the initial phase, and similar to IUPAC type III (indicating relatively weak interactions with the adsorbent). Generally, the maximum carbon dioxide adsorption coverage is only reached in the higher-pressure range. In the case of zeolites APD and AWO, methane adsorption coverage exceeds that of carbon dioxide at higher pressures. For these zeolites, the observed shape of the channel cross-section is closer to a circle, and this probably promotes a tighter arrangement of the spherical methane molecules in the pores (however, the effect does not necessarily apply if the gas phase contains both components). Another interesting phenomenon is exhibited by zeolite WEI: carbon dioxide adsorption dominates below 1000 kPa, while hydrogen sulfide adsorption becomes more significant above this pressure. This zeolite has one of the highest capacities for the acidic components, while methane adsorption amounts are nearly negligible. Another zeolite with a notably high adsorption capacity is zeolite CHA; however, methane adsorption is also significant in this case.

### 3.2. Mixture Adsorption

#### 3.2.1. Binary Mixture Adsorption

**Figure 2:**
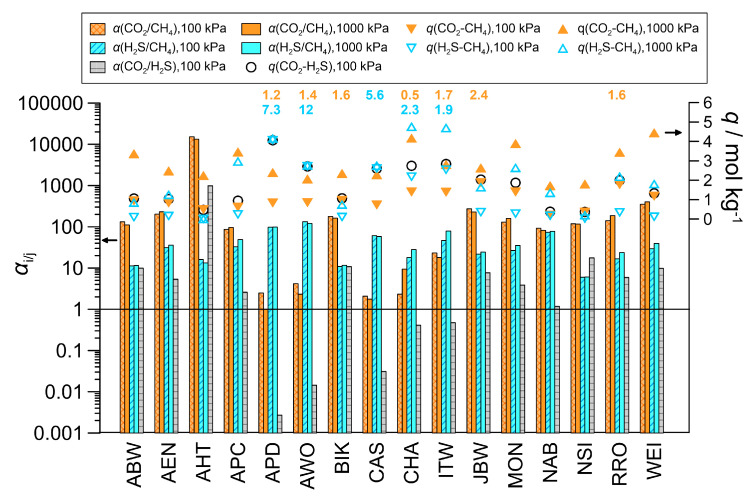
Total adsorption capacities (*q*, symbols) and selectivities (*α*, bars) for the equilibrium adsorption from the equimolar binary gas mixtures with the 16 selected zeolites at 298 K and at two different pressures (100 kPa and 1000 kPa). Numerical *α*/*α*_id_ values obtained at 298 K and 100 kPa are indicated above the corresponding bars (CO_2_/CH_4_: orange, H_2_S/CH_4_: blue).

Mixture adsorption simulations were performed on the 16 selected frameworks using equimolar binary gas mixtures of carbon dioxide and methane, hydrogen sulfide and methane, and carbon dioxide and hydrogen sulfide, all at 298 K. The results are shown in Figure 2. In addition to selectivities *α*, we also considered the adsorption capacities *q* of the zeolites. In several cases, the total adsorption capacities were low at 100 kPa, necessitating investigations at higher pressures. At 100 kPa, 11 zeolites prefer carbon dioxide and 5 zeolites (APD, AWO, CAS, CHA and ITW) prefer hydrogen sulfide, while methane adsorption remains low for most of them. In the case of the CO_2_-CH_4_ mixture, zeolite JBW shows the highest adsorption capacity for carbon dioxide, with a value of 1.86 mol/kg, but zeolites ITW, MON, RRO and WEI also have adsorption capacities greater than 1 mol/kg (which is often considered the lower limit for practical use). In the case of the H_2_S-CH_4_ gas mixture, zeolite APD shows the highest H_2_S adsorption capacity of 4 mol/kg. In terms of selectivity, the most effective separation of carbon dioxide and hydrogen sulfide can be achieved using zeolite AHT and zeolites APD and AWO, respectively. Zeolite AHT is also highly selective for carbon dioxide in the case of the CO_2_-H_2_S mixture. Zeolites CAS and CHA, previously studied by us [16], have been proven to be less selective for hydrogen sulfide than zeolites APD and AWO. At 1000 kPa, the higher partial pressure of the components in the gas phase creates a stronger driving force for adsorption. As a result, the capacities mostly reach the range that is relevant for industrial use. In the case of the CO_2_-CH_4_ gas mixture, the total adsorption capacities exceed 2 mol/kg in almost all cases. The highest CO_2_ capacity is achieved with zeolite WEI, obtaining a 3.5-fold increase as compared to the value at 100 kPa. Even zeolites with initially weaker adsorption performance also show a two- to threefold increase in CO_2_ adsorption. Of the APD, AWO and CAS frameworks, which exhibit lower CO_2_/CH_4_ loading ratios in the case of the pure components’ adsorption, zeolite APD exhibits approximately identical adsorption levels for the two gas components at 1000 kPa. No significant changes in CO_2_ selectivity can be observed when going from 100 to 1000 kPa; less than half of the zeolites tested produce a slight increase, while the rest show no change or a moderate decrease. At 1000 kPa, zeolite AHT reveals an acceptable adsorption capacity of 2.24 mol/kg and retains an outstanding selectivity of over 13,000. As several zeolites (e.g., APD, AWO, CAS) reach H_2_S saturation already in the lower pressure range, only a moderate capacity increase can be detected for these zeolites with the H_2_S-CH_4_ mixture at 1000 kPa. Increasing the pressure, the H_2_S selectivity also remains essentially unchanged for most of the zeolites studied or increased slightly for a few zeolites. Zeolites CAS and CHA, which were previously studied by us using the same potential models [16], produced essentially the same results as those obtained previously (which was both reassuring and expected). In another study, using slightly different model parameters, higher temperature and pressure, but the same gas composition [14], zeolites ABW, APD, AWO, BIK, JBW and WEI yielded similar selectivities (e.g., 70 instead of 124 for zeolite AWO, or 15 instead of 24 for zeolite JBW). Since an increase in temperature must result in a decrease in selectivity, this alone can explain why our results show slightly higher selectivities.

To verify that the exceptional CO_2_ separation performance was not due to the present modification of the potential model, simulations were also conducted using the unmodified TraPPE model for CO_2_. In the case of the CO_2_-CH_4_ gas mixture, the selectivity at 298 K and 100 kPa decreased only to ~10,000 and 100 with zeolite AHT and BIK, respectively. This means that the two potential models produce qualitatively similar results, i.e., the change required to more accurately reproduce the experimental CO_2_ isotherm on zeolite CHA does not significantly impact the identification of zeolites with good performance.

Comparing the selectivities with ideal selectivities (*α*_id_) can indicate the potential for competitive adsorption. The ideal selectivity, which is obtained when the uptake of one gas component is independent of that of the other component, was calculated from the adsorption isotherms by reading the loadings of the pure gas components at the pressures corresponding to the partial pressures of the components present in the binary mixtures. The *α*/*α*_id_ values (relative selectivities) calculated for the binary mixtures at 100 kPa are also shown in Figure 2 (data are only indicated when *α*/*α*_id_ < 0.8 or *α*/*α*_id_ > 1.2). Although several *α*/*α*_id_ values for the CO_2_-CH_4_ system remain around 1, in some cases (e.g., BIK, ITW, JBW, RRO) the mixture adsorption selectivity significantly exceeds the ideal value. In these cases, carbon dioxide effectively displaces methane from the adsorption sites. In the case of the H_2_S-CH_4_ mixture, exceptionally high *α*/*α*_id_ ratios can be detected for zeolites APD, AWO and CAS, where the real H_2_S selectivities exceed the ideal ones by 5–12 times. For these zeolites, the strong H_2_S preference is also evident in the low *α*/*α*_id_ values observed in the case of the CO_2_-H_2_S mixture (not indicated in Figure 2). Another good example of competition is that zeolite APD can bind 0.59 mol/kg of carbon dioxide from the CO_2_-CH_4_ mixture, but only 0.011 mol/kg from the CO_2_-H_2_S mixture. At 1000 kPa, where the zeolite pores are more densely filled, the relative selectivities were found to be significantly higher than 1 in all cases. This suggests once again that the acidic molecules have a higher affinity for the framework atoms.

**Figure 3:**
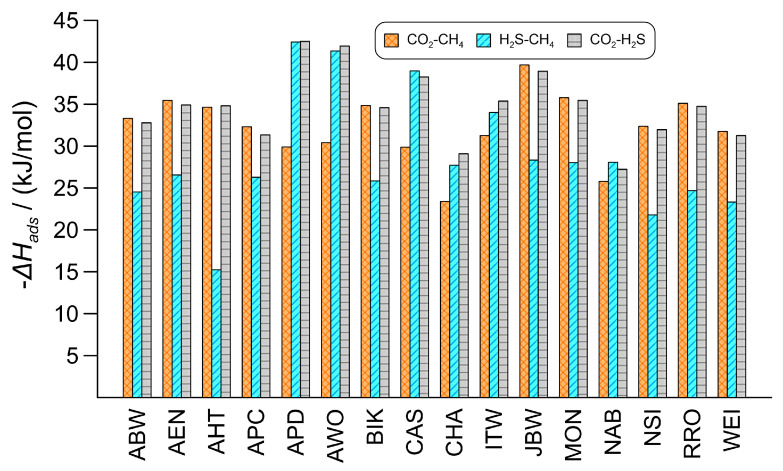
Heat of adsorption data for the equilibrium adsorption from the equimolar binary gas mixtures with the 16 selected zeolites at 298 K and 100 kPa.

The regeneration energy required by the adsorbent depends on the heat of adsorption. Equilibrium heat of adsorption shows the amount of energy released during the adsorption of a given component at equilibrium and provides information about the strength of interactions between the framework and the component. The difference between these quantities for two adsorbates also correlates with the corresponding selectivity: the greater the difference, the more favorable the separation. Figure 3 shows the total heat of adsorption values for the zeolites under investigation with the binary gas mixtures, demonstrating the total energy required for regeneration. The data are generally in the range of 20 to 40 kJ/mol, which is characteristic of physisorption. For the CO_2_-CH_4_ gas mixture, the total heat of adsorption values mostly fall within the range of 30 to 35 kJ/mol, and zeolite JBW exhibits the highest value (39.7 kJ/mol). For the H_2_S-CH_4_ gas mixture, the released heat is usually below 30 kJ/mol, but zeolites APD and AWO have exceptional values that exceed 40 kJ/mol. For these latter zeolites, the preferred component (H_2_S) is not only present in much larger quantities, but its heat of adsorption is also significantly higher.

#### 3.2.2. Ternary Mixture Adsorption

Adsorption simulations were performed for three-component gas mixtures consisting of methane, carbon dioxide and hydrogen sulfide, using a smaller group of promising IZA zeolites. The simulations were carried out at three different pressures (100, 1000 and 5000 kPa), two temperatures (298 K and 323 K), and with two gas compositions: an equimolar mixture (*y*_i_ = 0.333) and a gas mixture containing 95 mol% methane, 4 mol% carbon dioxide, and 1 mol% hydrogen sulfide, which somewhat represents natural gas. The group includes zeolite AHT, which appears to be the most effective for capturing carbon dioxide, and zeolites JBW, RRO and WEI, which are efficient at separating both acidic components from binary gas mixtures (although they tend to prefer carbon dioxide). Zeolite BIK is also present in this group; in a previous study [14], this zeolite exhibited a high affinity for carbon dioxide during the adsorption of a five-component mixture (H_2_S-CO_2_-CH_4_-C_2_H_6_-N_2_). As in [14], we also considered zeolites APD and AWO worthy of further investigation due to their high hydrogen sulfide uptake. The calculated selectivities and total adsorption capacities are listed in Table 3. At 100 kPa, most of the data remain below 1 mol/kg yet, but at higher pressures, the majority exceed this value. At 100 kPa, the selectivities for the equimolar ternary gas mixture are close to the corresponding values obtained for the equimolar binary gas mixtures; the small differences detected can be attributed to the slightly different partial pressures of the components in the binary and ternary mixtures. This suggests that the interaction between the two acidic components in the adsorption phase does not significantly influence the loading ratios, at least at lower pressures. At 100 kPa, there is still enough free space in the pores so that the presence of hydrogen sulfide does not significantly promote (or inhibit) carbon dioxide binding, and vice versa. In relation to this, the observation can also be made that in the case of the gas mixture mimicking the composition of natural gas, generally small decreases or insignificant changes in selectivity occur at both temperatures compared to the corresponding values of the equimolar gas mixture.

In many cases, the maximum selectivity is achieved at 1000 kPa. At 5000 kPa, a substantial increase in adsorption driving forces probably compensates for differences in component affinity to the framework to some extent, and this reduces selectivity. The best CO_2_ separation is achieved by zeolite AHT, and zeolite WEI probably has the best overall separation performance. The table clearly shows the large negative effect of an increase in temperature; however, this effect is less pronounced for the capacities at higher pressures. This reduction in selectivity is due to the fact that the adsorption of pure carbon dioxide or hydrogen sulfide is accompanied by a higher heat of adsorption, and raising the temperature shifts the adsorption equilibrium towards higher methane loadings. For all these reasons, we conducted a shorter test at 273 K and 5000 kPa, using the gas mixture with the more realistic composition. This additional test produced significantly higher selectivities in all cases, ranging from 50% to 100% more than the corresponding results at 298 K. Therefore, if other considerations permit, a slightly lower-temperature separation process can also be considered in practice.

**Figure 4:**
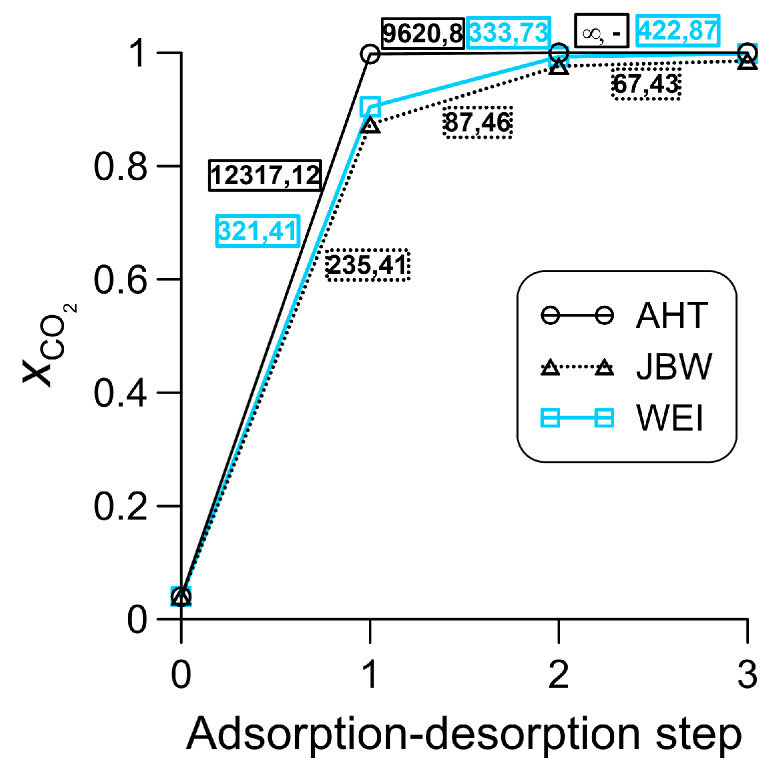
Mole fractions of carbon dioxide in the adsorption phase during a multi-step adsorption–desorption test performed at 298 K and 5000 kPa (in step 0, the mole fraction of the initial gas phase is shown). Selectivity numbers for the individual steps are also indicated; the first and second numbers always represent the CO_2_ and H_2_S selectivity, respectively.

A multi-step adsorption–desorption test was also carried out with zeolites AHT, JBW and WEI, which were found to be most suitable for carbon dioxide separation, at 298 K and 5000 kPa, using a gas mixture that mimics the composition of natural gas. This test indirectly models the adsorption purification of natural gas (through the enrichment of contaminants) using an idealized process. Idealized circumstances imply 100% regeneration after the equilibrium adsorption step, after which the resulting gas comes into contact with the clean adsorbent. Furthermore, it is assumed that the quantity of gas obtained by desorption will always be sufficient to ensure that the change in driving force is negligible during the next equilibrium adsorption step. The results of the investigation are shown in Figure 4. In the first adsorption step, the mole fraction of adsorbed carbon dioxide consistently exceeds 0.8. By the end of the third adsorption step, this value increases to at least 0.99 with zeolites JBW and WEI, whereas no methane and hydrogen sulfide bindings occur with zeolite AHT throughout the entire simulation length in the third adsorption step. The corresponding selectivities are also indicated in Figure 4. It is worth considering the CO_2_ selectivities in the first two steps, as these steps involve the greatest differences in input composition. For zeolites AHT and JBW, there are substantial decreases with increasing carbon dioxide inlet concentration, whereas there are no significant changes for zeolite WEI. At the same time, the H_2_S selectivity typically remains unaffected, and in the case of zeolite WEI, it even increases as the adsorption–desorption cycles progress.

### 3.3. Structural Relationships

**Figure 5:**
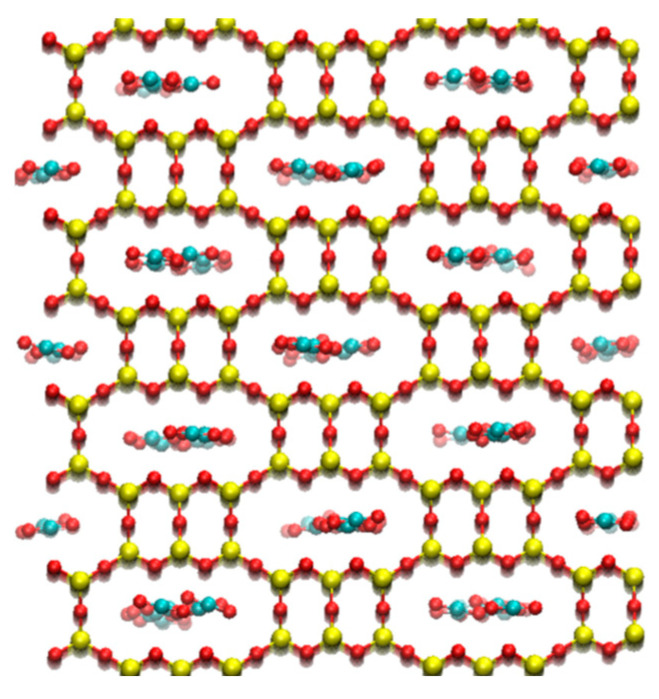
Illustration of the characteristic orientations of the CO_2_ molecule in the pores of zeolite AHT (O: red; Si: yellow; C: cyan).

Analysis of the simulation snapshots revealed that the various gas components mostly favor different adsorption sites within the zeolites, at least at ambient pressure. However, it was found that methane and hydrogen sulfide molecules can bind to similar sites, where roughly spherical free spaces are available for adsorption. When focusing on carbon dioxide, it was observed that the position of carbon dioxide molecules in the selected frameworks is usually not random. In the relevant adsorption pores, the molecule is typically arranged such that its central C atom is positioned between the central O atoms of two opposing Si-O-Si atom groups, the Si-O-Si bond angles of which are greater than 180° (when viewed from the C atom; this means that these O atoms are in the closest possible position to each other). If the positions of these O atoms in the framework are favorable, the CO_2_ molecule can bind in relatively narrow pores, provided that such small distances between the opposite zeolite O atoms afford sufficient space for the O atoms of the linear CO_2_ molecule, too. Our simulations showed that carbon dioxide is adsorbed even in zeolites where the pore diameter is smaller than that previously found to be favorable for the efficient binding of hydrogen sulfide. Figure 5 also illustrates that these pores can have a slightly flattened cross-section. Due to differences in the preferred adsorption sites, competition between carbon dioxide and methane is generally not significant at lower pressures for the zeolites studied. However, zeolite JBW is a notable exception. Here, the cross-section of the pores is more rounded, and the LCD parameter is relatively small; competitive adsorption is therefore governed by stronger interactions between CO_2_ molecules and framework atoms. In contrast, the relatively large free space in zeolite CHA (as evidenced by the substantial LCD parameter) counteracts selectivity.

Regarding the relationship between framework geometry and CO_2_ selectivity, one of the critical factors is the PLD parameter of the zeolites: there is a lower limit to the PLD value below which molecules cannot enter the framework. Consequently, it would be reasonable to assume that there is a close correlation between the PLD parameter and selectivity, given the slightly different sizes of the gas molecules under investigation. In fact, however, we could only observe a very weak connection between an increase in the PLD parameter and a decrease in CO_2_ selectivity.

**Figure 6:**
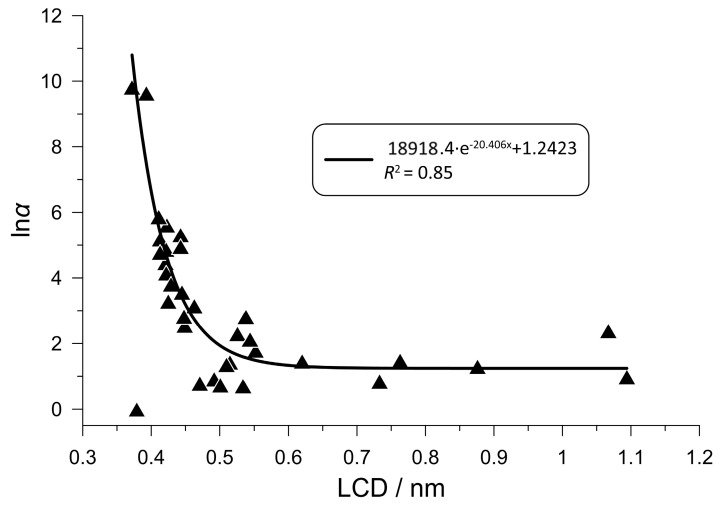
LCD parameters of the originally selected IZA zeolites and their corresponding CO_2_ selectivities for the equilibrium adsorption from the CO_2_-CH_4_ gas mixture at 298 K and 100 kPa. An exponential function was fitted to the data, which approximately describes the possible relationship.

It was found that the LCD parameter is a more important factor. Figure 6 shows the correlation between the LCD parameter and CO_2_ selectivity at 298 K and 100 kPa for more than 30 zeolites originally chosen for further study. There is a narrow range of LCD parameters below which no carbon dioxide adsorption can be observed, and within which CO_2_ selectivity increases sharply as the parameter decreases. Although the LCD parameter is generally only loosely proportional to the PLD parameter for the zeolites under investigation, the critical LCD value below which the adsorption capacity becomes practically zero is obviously related to the PLD parameter. Furthermore, we experienced a significant decrease in adsorption capacity as the LCD parameter decreased. Zeolites AHT and MVY, e.g., have the lowest LCD values (0.393 and 0.372 nm, respectively) and show the highest CO_2_ selectivities, but their adsorption capacities are below average (0.08 mol/kg for zeolite MVY). It was also detected that zeolites with lower LCD values (e.g., ABW, AEN, AHT, BIK, JBW and NSI) tend to prefer carbon dioxide, whereas zeolites with higher LCD values (e.g., APD, AWO, CAS and CHA) tend to prefer hydrogen sulfide. Previous studies [16,17] have shown that density has some predictive power, too: zeolites with a higher density and, consequently, a more compact pore structure generally exhibit greater CO_2_ or H_2_S selectivity in gas mixtures containing methane.

In conclusion, it seems that predictions of selectivity based solely on structural mapping rather than atomistic simulations can be inaccurate at this stage, since selectivity depends on the combined effect of several complex geometric factors (other studies have also failed to make significant breakthroughs in this respect so far). However, the LCD parameter appears to be suitable for qualitatively predicting the adsorption separation performance on an empirical basis.

### 3.4. Theoretical Zeolites with a High Preference for Carbon Dioxide

Based on the above, our final empirical correlation equation constructed for the CO_2_ selectivity also considers the PLD parameter and the framework density, but assigns them smaller weights:

*Y* = ln(LCD^4^ ∙ PLD^0.1^ ∙ density^−0.5^) + 7.58. (4)

The constant in this equation is responsible for shifting the favorable range around zero; the function returns with a *Y* value between −0.3 and +0.3 for the promising IZA zeolites.

**Figure 7:**
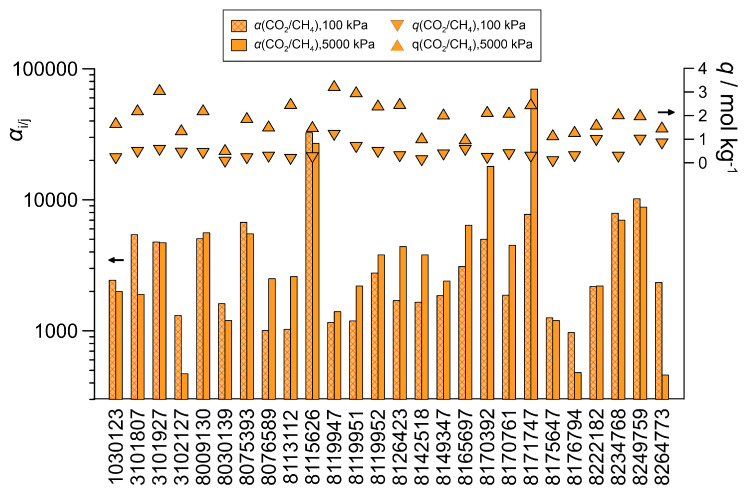
Total adsorption capacities (*q*, symbols) and CO_2_ selectivities (*α*, bars) for the equilibrium adsorption from the equimolar CO_2_-CH_4_ gas mixtures with promising theoretical zeolites at 298 K and at two different pressures (100 kPa and 5000 kPa).

Applying Equation (4), the analysis was extended to theoretical frameworks. Theoretical zeolites are designed using computer-aided structure generation and have not yet been synthesized in laboratories. There are a huge number of stable hypothetical pure silica zeolites [9,10], and within these, a database from our previous study [17], containing only 4915 potentially synthesizable frameworks, was used (the selection was mainly based on the so-called local interatomic distances criteria [12]). In our study [17], more than 20 frameworks with a high preference for hydrogen sulfide over methane were identified in this database. By filtering with the above equation, ~180 potentially suitable zeolites were detected, and such a relatively small group could now be handled by simulations with the equimolar CO_2_-CH_4_ gas mixture. Figure 7 summarizes the theoretical pure silica structures for which the CO_2_ selectivity exceeds 1000 at 298 K and 100 kPa. As the adsorption capacity was not satisfactory for most of these frameworks, simulations were also performed at 5000 kPa. At this pressure, the adsorption loadings generally exceed 1 mol/kg. While the increase in pressure often led to a decrease in selectivity, it also resulted in a significant increase for several zeolites (e.g., a tenfold increase either in selectivity or in capacity with zeolite 8171747). Of the structures identified as promising by Equation (4), 65% exhibited selectivity above 50, with nearly 15% achieving values above 1000 at 100 kPa. This limitation emphasizes that Equation (4) is designed for qualitative screening purposes and should be interpreted with caution. While the examined structural parameters and this empirical equation appear to be appropriate for identifying a manageable number of potentially suitable frameworks, molecular simulations are essential for accurately predicting adsorption performance. The frameworks deemed suitable in the simulations did not exhibit any distinctive features; in other words, the ineffective structures did not differ significantly from the other high-performing ones. As was preliminarily assumed, this implies that selectivity must also be governed by other, as yet unidentified, complex geometric factors. Nevertheless, studying theoretical zeolites using computer experiments enables us to explore further possible correlations between structural properties and adsorption behavior, as it allows us to examine a much larger number of frameworks than is possible with IZA zeolites.

## 4. Conclusions

The adsorption performance of pure silica zeolites was investigated using molecular simulations, focusing on the separation of the main acidic contaminants of natural gas, carbon dioxide and hydrogen sulfide, from methane. A step-by-step reduction method was employed. First, pre-screening was performed on more than 260 available idealized/optimized IZA zeolite structures using the PoreBlazer structure analysis program. Then, 37 IZA zeolite candidates were selected, for which adsorption isotherms and ambient-pressure binary mixture adsorption data were determined at 298 K. After that, the test group of zeolites was narrowed down to 16 frameworks, and various mixture adsorption simulations were carried out with them, examining the adsorption of the possible binary gas mixtures with equimolar compositions. Based on these results, seven promising zeolites were chosen for further investigation involving three-component gas mixtures at different pressures, temperatures, and gas compositions.

In terms of practical applicability, the IZA zeolites AHT, APD, AWO, JBW and WEI proved to be the most promising. Zeolite AHT, with its particularly high CO_2_ selectivity, can be an excellent candidate for purifying natural gas streams containing relatively large amounts of carbon dioxide and negligible amounts of hydrogen sulfide. Zeolite WEI may be suitable for effectively removing both acidic components from natural gas. In this respect, zeolite JBW is not much worse, and zeolites ABW, AEN, APC, BIK, MON, NAB and RRO are not far behind it either. As these zeolites primarily prefer carbon dioxide, the separation shifts towards enhanced carbon dioxide removal in the case of a multi-step adsorption process. In terms of operating costs, zeolite WEI has one of the lowest heat of adsorption values at 298 K and 100 kPa and one of the highest adsorption capacity values at 298 K and 5000 kPa. For natural gas sources that are relatively rich in hydrogen sulfide, the most suitable pure silica IZA candidates would be zeolites APD and AWO (and not the previously identified CAS framework [16]). Regarding the adsorption conditions of the examined zeolites, it was observed that increasing the pressure often leads to a decrease in selectivity, although the effect varies. Increasing the temperature, in addition to the expected reduction in adsorption capacity, always leads to a significant decrease in selectivity. This is consistent with the exothermic nature of adsorption and the higher heat of adsorption values of the acidic components compared to those of methane.

As previously established [17], some of the structural parameters provided by the PoreBlazer structure analysis software correlate with selectivity trends. According to the present analysis, the LCD parameter was found to be the most decisive factor in the CO_2_ separation performance of the pure silica IZA zeolites. Within certain limits, it can be concluded that structures with lower LCD values prefer carbon dioxide, while those with higher values prefer hydrogen sulfide. Examining a relatively large number of IZA zeolites enabled an empirical equation to be constructed based on structural properties in order to predict CO_2_ selectivity. This enabled us to efficiently screen a database containing thousands of theoretical, yet potentially synthesizable, pure silica zeolites, resulting in a significantly smaller group of structures. Using molecular simulations with this group of zeolites, nearly 30 theoretical frameworks with an exceptionally high CO_2_ selectivity were identified.

Finally, it should be noted that the obtained simulation results are, of course, dependent on the accuracy of the atomic interaction models employed. Compared to the limited experimental results available, good performance is demonstrated by the interaction models used here, but improving their predictive power can be a constant goal. Currently, the identification of pure silica zeolite structures suitable for specific separation tasks is hampered by the lack of available equilibrium adsorption experimental results. At the same time, it cannot be ignored that the present simulation estimates were made with error-free, idealized/optimized zeolite structures. When making future comparisons with the expected new experimental data, the possible effects of structural defects, intergranular spaces and contaminants in real zeolite materials must be taken into account.

## Figures and Tables

**Table 1:** Lennard-Jones energy (*ε*) and size (*σ*) parameters and partial charges (*Q*) belonging to the atomic interaction centers in the applied potential models (*k*_B_ is the Boltzmann constant and *e* is the elementary charge).

Center	*ε*/*k*_B_/K	*σ*/nm	*Q*/*e*	Position in the Molecule
O	53.0	0.330	−0.75	experimental atomic
Si	22.0	0.230	1.50	experimental atomic
S (H_2_S)	270.0	0.376	−1.152	S-H distance: 0.1348 nm
H (H_2_S)	0	0	0.268	H-S-H angle: 91.61°
X (H_2_S)	0	0	0.308	S-X distance: 0.08764 nm
				X-S-X angle: 110.0°
C (CH_4_)	80.0	0.340	−0.660	C-H distance: 0.1090 nm
H (CH_4_)	7.901	0.265	0.165	H-C-H angle: 109.47°
C (CO_2_)	45.0	0.280	0.70	C-O distance: 0.1160 nm
O (CO_2_)	79.0	0.305	−0.350	O-C-O angle: 180.0°

**Table 2:** Some properties of the IZA zeolites examined. *α* is the selectivity obtained from adsorption simulation with the CO_2_-CH_4_ binary mixture at 298 K and 100 kPa (the ‘symmetry’ data are provided for supplementary crystallographic information only).

Zeolite	PLD/nm	LCD/nm	Density kg/m^3^	Symmetry	*α* (CO_2_/CH_4_)
ABW	0.347	0.418	1755	orthorhombic	132
ACO	0.355	0.449	1643	cubic	13
AEN	0.350	0.443	2008	orthorhombic	202
AHT	0.278	0.393	1917	orthorhombic	15,255
APC	0.315	0.421	1765	orthorhombic	87
APD	0.369	0.492	1795	orthorhombic	2.5
ATT	0.368	0.538	1704	orthorhombic	17
ATV	0.343	0.471	1887	orthorhombic	2.2
AWO	0.364	0.515	1819	orthorhombic	4.2
BCT	0.285	0.379	1895	tetragonal	-
BIK	0.335	0.413	1862	orthorhombic	178
BOF	0.378	0.552	1823	orthorhombic	6.0
BRE	0.294	0.526	1827	monoclinic	10
CAS	0.307	0.501	1871	orthorhombic	2.1
CHA	0.355	0.733	1502	trigonal	2.3
CZP	0.344	0.425	2126	hexagonal	27
EPI	0.360	0.544	1764	monoclinic	8.3
ESV	0.319	0.620	1771	orthorhombic	4.3
EWO	0.378	0.534	1902	orthorhombic	2.0
GOO	0.307	0.448	1892	orthorhombic	17
ITW	0.375	0.463	1768	monoclinic	23
JBW	0.317	0.423	1874	orthorhombic	272
JSN	0.338	0.510	1783	monoclinic	3.9
KFI	0.411	1.067	1494	cubic	11
LTA	0.422	1.094	1414	cubic	2.7
LTJ	0.306	0.408	1850	tetragonal	1.0
MEL	0.514	0.763	1732	tetragonal	4.4
MON	0.334	0.422	1761	tetragonal	130
MVY	0.279	0.372	2093	orthorhombic	18,286
NAB	0.339	0.429	1605	tetragonal	92
NPO	0.373	0.422	1867	hexagonal	63
NSI	0.292	0.413	1874	monoclinic	118
RRO	0.400	0.443	1782	monoclinic	141
UWY	0.610	0.876	1627	orthorhombic	3.7
VSV	0.322	0.429	1676	tetragonal	45
WEI	0.340	0.411	1647	orthorhombic	352
YUG	0.315	0.445	1791	monoclinic	35

**Table 3:** Total adsorption capacities (*q*) and selectivities (*α*) for the selected 7 zeolites obtained from gas adsorption simulations involving ternary mixtures of different compositions (*y*) at various temperatures and pressures.

Zeolite	Temperature Composition	*α*^100 kPa^ (CO_2_/CH_4_)	*α*^100 kPa^ (H_2_S/CH_4_)	*q*^100 kPa^ mol/kg	*α*^1000 kPa^ (CO_2_/CH_4_)	*α*^1000 kPa^ (H_2_S/CH_4_)	*q*^1000 kPa^ mol/kg	*α*^5000 kPa^ (CO_2_/CH_4_)	*α*^5000 kPa^ (H_2_S/CH_4_)	*q*^5000 kPa^ mol/kg
AHT	298 K (*y*_CH_4__ = 0.33)	16,880	16	0.334	14,550	17	1.973	11,190	11	2.570
	323 K (*y*_CH_4__ = 0.33)	6960	12	0.111	6390	11	1.009	5650	11	2.182
	298 K (*y*_CH_4__ = 0.95)	16,250	13	0.038	14,560	15	0.383	12,320	12	1.352
	323 K (*y*_CH_4__ = 0.95)	9180	19	0.013	6730	11	0.131	5780	10	0.543
BIK	298 K (*y*_CH_4__ = 0.33)	175	15	0.770	191	27	2.327	126	25	2.753
	323 K (*y*_CH_4__ = 0.33)	102	10	0.315	115	16	1.686	101	16	2.473
	298 K (*y*_CH_4__ = 0.95)	154	11	0.118	163	15	0.873	169	19	1.908
	323 K (*y*_CH_4__ = 0.95)	94	9	0.045	104	11	0.400	100	12	1.210
JBW	298 K (*y*_CH_4__ = 0.33)	306	33	1.758	256	55	2.754	168	49	2.971
	323 K (*y*_CH_4__ = 0.33)	166	20	0.829	166	31	2.389	122	31	2.811
	298 K (*y*_CH_4__ = 0.95)	275	23	0.369	300	36	1.812	235	41	2.488
	323 K (*y*_CH_4__ = 0.95)	161	17	0.131	168	20	0.961	156	23	2.010
RRO	298 K (*y*_CH_4__ = 0.33)	135	22	1.488	195	37	3.400	158	35	3.724
	323 K (*y*_CH_4__ = 0.33)	72	14	0.599	103	22	2.749	96	20	3.468
	298 K (*y*_CH_4__ = 0.95)	111	17	0.215	136	23	1.645	149	26	2.967
	323 K (*y*_CH_4__ = 0.95)	69	13	0.089	73	14	0.751	82	16	2.113
WEI	298 K (*y*_CH_4__ = 0.33)	344	34	0.867	361	63	4.336	304	67	5.588
	323 K (*y*_CH_4__ = 0.33)	199	23	0.346	211	32	2.761	184	40	4.790
	298 K (*y*_CH_4__ = 0.95)	334	29	0.106	335	33	0.975	321	41	3.096
	323 K (*y*_CH_4__ = 0.95)	195	21	0.042	198	22	0.407	186	23	1.620
APD	298 K (*y*_CH_4__ = 0.33)	0.35	99	3.990	0.20	99	4.144	0.19	71	4.156
	323 K (*y*_CH_4__ = 0.33)	0.62	60	3.410	0.21	63	4.098	0.16	49	4.145
	298 K (*y*_CH_4__ = 0.95)	2.3	62	1.029	0.46	89	3.731	0.22	74	4.061
	323 K (*y*_CH_4__ = 0.95)	2.2	39	0.396	0.85	52	2.845	0.32	51	3.864
AWO	298 K (*y*_CH_4__ = 0.33)	2.0	135	2.680	2.0	145	2.764	1.7	132	2.772
	323 K (*y*_CH_4__ = 0.33)	1.9	78	2.388	1.5	80	2.737	1.4	62	2.764
	298 K (*y*_CH_4__ = 0.95)	3.9	91	0.931	2.0	117	2.525	1.7	100	2.712
	323 K (*y*_CH_4__ = 0.95)	3.5	55	0.381	2.0	69	2.056	1.4	64	2.608

## Data Availability

Dataset available on request from the authors.

## Abbreviations

The following abbreviations are used in this manuscript:

IZA	International Zeolite Association
PLD	Pore limiting diameter
LCD	Largest cavity diameter
DSLF	Dual-Site Langmuir-Freundlich
IUPAC	International Union of Pure and Applied Chemistry
